# Serotonergic gene polymorphisms (5-*HTTLPR*, 5*HTR1A*, 5*HTR2A*), and population differences in aggression: traditional (Hadza and Datoga) and industrial (Russians) populations compared

**DOI:** 10.1186/s40101-018-0171-0

**Published:** 2018-04-16

**Authors:** Marina L. Butovskaya, Polina R. Butovskaya, Vasiliy A. Vasilyev, Jane M. Sukhodolskaya, Dania I. Fekhredtinova, Dmitri V. Karelin, Julia N. Fedenok, Audax Z. P. Mabulla, Alexey P. Ryskov, Oleg E. Lazebny

**Affiliations:** 1 Department of Cross-Cultural Psychology and Human Ethology, Institute of Ethnology and Anthropology, Russian Academy of Sciences, Leninsky Ave. 32a, Moscow, Russia 119334; 2 Faculty of History, Lomonosov Moscow State University, Lomonosovsky Ave. 27-4, Moscow, Russia 119192; 3 Russian State University for the Humanities, Miusskaya Sq. 6, GSP-3, Moscow, Russia 125993; 4 Group for Population Immunogenetics, Vavilov Institute of General Genetics, Russian Academy of Sciences, Gubkina St. 3, Moscow, Russia 119333; 5 Department of Genome Organization, Institute of Gene Biology, Russian Academy of Sciences, Vavilova St. 34/5, Moscow, Russia 119334; 6 Faculty of Biology, Lomonosov Moscow State University, Leninskie Gory 1-12, Moscow, Russia 119991; 7 Department of Archaeology and Heritage, University of Dar es Salaam, P.O. Box 35091, Dar es Salaam, Tanzania; 8 Department of Evolutionary and Developmental Genetics, Koltzov Institute of Developmental Biology, Russian Academy of Sciences, Vavilova St. 26, Moscow, Russia 119334

**Keywords:** 5-*HTTLPR*, 5*HTR1A*, 5*HTR2A*, Total aggression, Hadza, Datoga, Russians

## Abstract

**Background:**

Current knowledge on genetic basis of aggressive behavior is still contradictory. This may be due to the fact that the majority of studies targeting associations between candidate genes and aggression are conducted on industrial societies and mainly dealing with various types of psychopathology and disorders. Because of that, our study was carried on healthy adult individuals of both sex (*n* = 853).

**Methods:**

Three populations were examined: two traditional (Hadza and Datoga) and one industrial (Russians), and the association of aggression with the following polymorphisms 5-*HTTLPR,* rs6295 (5*HTR1A* gene), and rs6311 (5*HTR2A* gene) were tested. Aggression was measured as total self-ratings on Buss-Perry Aggression Questionnaire.

**Results:**

Distributions of allelic frequencies of 5-*HTTLPR* and 5*HTR1A* polymorphisms were significantly different among the three populations. Consequently, the association analyses for these two candidate genes were carried out separately for each population, while for the 5*HTR2A* polymorphism, it was conducted on the pooled data that made possible to introduce ethnic factor in the ANOVA model. The traditional biometrical approach revealed no sex differences in total aggression in all three samples. The three-way ANOVA (μ + *5-HTTLPR* + 5*HTR1A* + 5*HTR2A* +ε) with measures of self-reported total aggression as dependent variable revealed significant effect of the second serotonin receptor gene polymorphism for the Hadza sample. For the Datoga, the interaction effect between *5-HTTLPR* and 5*HTR1A* was significant. No significant effects of the used polymorphisms were obtained for Russians. The results of two-way ANOVA with ethnicity and the 5*HTR2A* polymorphism as main effects and their interactions revealed the highly significant effect of ethnicity, 5*HTR2A* polymorphism, and their interaction on total aggression.

**Conclusions:**

Our data provided obvious confirmation for the necessity to consider the population origin, as well as cultural background of tested individuals, while searching for associations between genes and behavior, and demonstrated the role of cultural attitudes towards the use of in-group aggression. Our data partly explained the reasons for disagreement in results of different teams, searching for candidate-gene associations with behavior without considerations of culturally desirable norms. Previous studies suggested that the 5*HTR2A* gene polymorphism associates with aggression and criminality. Our data extended these findings, demonstrating the role of rs6311 (5*HTR2A* gene) in aggression in adult healthy men and women from our samples. We found that *G*-allele carriers were rated higher on total aggression.

## Background

Socio-psychological research underscores the relation between cognition, emotion, and aggression. It is documented that negative effect such as fear and anxiety frequently precipitates and modulates aggressive behavior, particularly reactive [1]. The current knowledge on genetic basis of aggression is contradictive [2–4]. The conclusions of these studies were generalized to all human populations, without taking into account cultural differences and cross-cultural comparisons.

For decades, it was a dogma that the neurotransmitter serotonin (5-HT) has been implicated in the modulation of aggression in both animals and humans, and that aggression and serotonergic activity are inversely related [5, 6]. With growing number of studies, it becomes obvious that different types of aggression are differentially regulated by the 5-HT system and different 5-HT receptors seem to be involved [7]. Complex bidirectional relationships between aggression and serotonin, based on the interplay between impulse controls and social functioning were demonstrated [8]. Serotonin affects psychological characteristics and social interactions that have an impact on violent behavior; in turn, the psychological and social factors exert an influence on serotoninergic function [8].

It is suggested that predisposition to aggression appears to be deeply affected by the polymorphic genetic variants of the serotoninergic system. Candidate gene studies have found associations mainly with dopaminergic and serotonergic genes (*MAOA. 5HTT*, *HTR1B*, *HTR2A*, *DAT*, *DRD2*, *DRD4*) and with hormone-related genes (*AR*, *ESR1*, *AVP*, *OXTR*), which have historically received most attention (see [9] for detailed discussion). A number of candidate genes associated with serotoninergic system functioning were subjected for testing. Among these candidates, the serotonin transporter (5-HTT) was selected as primary candidate due to the relationship between these polymorphic variants and anatomical changes in the limbic system of aggressive people [2, 10]. The reduced 5-HTT availability in the cingulate gyrus was reported in aggressive subjects, thus suggesting that serotonin metabolism in this structure may be a crucial determinant of disposition towards violent behavior [11].

An association between short 5-*HTTLPR* (*S*) allele and violent behavior has been demonstrated in a number of studies [12–16]. The long genetic variant (*L*) associated with enhanced gene expression; it was demonstrated that the uptake of 5-HT is up to 50% less in cells carrying one or two copies of the *S* allele than in cells homozygous for the *L* allele. The short allele is dominant and results in decreased concentration of the transporter protein and a poorer response to stressful events [17]. In adults, most of studies indicated that the short variant of 5-*HTTLPR* driving lower transcription of genes was associated with aggression, anger, hostility violence, and criminality [4]. Certain studies suggest that the genotype *SS* exhibited the highest aggression tendencies in humans [18, 19].

Another gene candidates associated with aggression are various genes of serotoninergic receptors. Witte and others [20] pointed to a higher density of inhibitory 5-HT1A receptors in frontal areas in subjects exhibiting higher aggression scores and [21] obtained similar results for rats. The 5-HT1A receptors subtype may be critical for depression, as they have high density in limbic and cortical regions involved in mood regulation [22, 23]. Same authors stated that a functional polymorphism in the *5-HTT* gene, but not the 5*HTR1A* gene, affects 5-HT1A receptor availability in man. According to data presented in review [18], the carriers of the *G* allele of the 5*HTR1A* gene are less aggressive and more sensitive to depression and neuroticism, while carriers of the homozygous *CC* genotype demonstrated association with aggression in patients with Alzheimer disease.

The *5HTR2A* receptor gene has been also mentioned as a gene candidate tested in relation with functioning of serotoninergic system. The 5-HT2A receptor is the most abundantly expressed serotonin receptor subtype in the cortex, and it is predominantly expressed in pyramidal neurons [24–26]. It has been functionally and genetically associated with schizophrenia, autism, attention-deficit/hyperactivity disorder, and affective disorders [27–30]. *GG* genotype of rs6311 (*5HTR2A* gene) were found to be associated with anger and aggression-related behavior in Germans [31]. The association between the *5HTR2A* and impulsivity has been recently demonstrated by Japanese team [32], while Hungarian scholars analyzed 55 SNPs located in the *5HTR2A*, and found that only one SNP, rs7322347, appeared significantly associated with three subscales of Buss and Perry’s Aggression Questionnaire (BPAQ), namely physical aggression, anger, and hostility [3].

The majority of studies targeting associations between serotonergic genes polymorphisms and aggression were conducted on industrial societies and (or) were mainly dealing with various types of psychopathology and disorders. Still, to understand the general trends in association of serotoninergic genes polymorphisms with individual’s aggressiveness, the cross-cultural study including representatives of traditional and industrial populations may be of primary importance. In this case, the understanding of general peculiarities of interactions between the ecological and social patterns may be crucial. The good example may be found in socioecological theories, linking social structure (egalitarian vs despotic) with the levels of intragroup contest competition in connections to severity of aggression within group members in primates [33]. The degree of sex differences in aggression, particularly, physical, may vary cross-culturally in humans [34]. Primate models interpreting the influence of social structure in levels of contest competition and sex differences in aggression deserve particular attention in this respect.

The goals of current study were to assess the association of sex and three genes of the serotoninergic system (serotonin transporter, *5-HTT*, and two receptors of serotonin, *5HTR1A* and *5HTR2A*) with total aggression based on self-ratings on BPAQ in representatives of three human populations: two rural traditional African groups, Hadza and Datoga, and one urban sample from Russia, represented by Russian students.

Our sample selection was not by chance. Hadza are traditional nomadic egalitarian foragers with the low level of aggressiveness [35–37]. Datoga being semi-nomadic pastoralists, known formerly to be engaged in interethnic cattle raiding with Maasai and Sukuma, are known for higher level of aggression [35, 37–39]. Sample of Russian students was selected for comparison because we expected that aggression in this group have been controlled by rigid cultural norms and social attitudes, typical for modern European societies. Besides, given the fact that our particular Russian sample was composed of students, we suggested that the level of total aggression would be even lower compared to population mean.

The following predictions were tested:

1. The study groups will differ by the level total aggression, based on self-ratings on BPAQ.
2. Sex difference in total aggression will be observed in Datoga, in contrast to two other samples, given more patriarchal character of Datoga society.
3. Differences in allele frequency distributions for the all three polymorphisms, selected in this study among the study samples, are expected.
4. The Sa+ genotypes of the 5-*HTTLPR* polymorphism will be associated with higher scores on total aggression within each sample.
5. The *GG* genotype of *5HTR1A* polymorphism will be associated with higher scores on total aggression within each sample.
6. The *GG* genotype of *5HTR2A* polymorphism will be associated with higher scores on total aggression within each sample.
7. The interaction effect of the three polymorphisms studied are expected to be significant in association with higher aggression in the studied samples.

## Methods

### Participants

Data on 853 individuals (mean age: 28.9 ± 13.5 years; age range: 15–80 years) from three populations were obtained (Table 1). The data on Hadza and Datoga were collected in the Mangola region of Northern Tanzania. The genetic samplings were conducted in 2006–2007, and in the period from 2006 to 2014 for the same individuals, the demographic data and self-ratings on aggression were collected. The identical data on students from Russian were collected in 2010 and 2013. Consequently, two study groups were represented by traditional people, illiterate in majority, living in rural environment, with age range 15–80 years (for more information on Hadza and Datoga samples, see [36–38]). Russian University students with the age range 16–28 years represented the last sample (see Table 1).

**Table 1:** Population origin and sex of participants

Population	Men	Women	Total
Hadza	187	142	329
Datoga	162	157	319
Russians (students)	86	119	205

We secured the age of African participants on the bases of a calendar of well-dated and memorable events in local history, as the majority of Hadza and Datoga are not literate and do not keep birth records. Each participant was assigned to a 10-year age group (1 = 16–19 years; 2 = 20–29 years; 3 = 30–39 years; 4 = 40–49 years; 5 = 50–59 years; 6 = 60+ years) following previous protocols [40].

### Protocols

All African participants (Hadza and Datoga) were interviewed in Swahili by the first author or a trained local assistant. They were asked to provide information including their age, sex, marital status, ethnicity, and aggression. All questions were read aloud in one-to-one dialogues and further explanations were provided, if necessary. Self-reported aggression was assessed with the BPAQ [41], based on 29 statements, grouped into four subscales—physical aggression (9 items), verbal aggression (5 items), anger (7 items), and hostility (8 items)—answered on a Likert scale anchored by 1 (extremely uncharacteristic of me) and 5 (extremely characteristic of me). The translation of the BPAQ into Swahili was done previously, following accepted standards (translation and back translations by four bilingual assistants [42, 43]. More details about the procedure of data collection may be found in our previous papers [37, 44].

The procedure of data collection for Russian students was different, as they were able to fill in the questionnaire, as well as demographic data individually. Russian students were working with the Russian version of BPAQ, which was initially translated and validated by us, and already applied in a number of previous studies [45]. All data were collected anonymously, to meet the ethical demands.

Scores on aggression were calculated only for respondents who answered all items. The data were obtained for all four scales of BPAQ (29 questions). Because verbal aggression was considered acceptable in all the three study groups, we considered important to include the ratings on this subscale as well. For the purposes of this paper, we targeted the total aggression (represented as sum of these scales), because we were intended to target aggression per se. In doing this, we account for possible cultural limitations (particularly, cultural restrictions in applications of physical aggression, anger, and hostility).

### Participants

The Hadza and the Datoga participants were personally interviewed to determine their age, sex, ethnicity, and personal history, as well as questions on aggression, based on BPAQ [41] as described previously [37, 44]. All interviews were conducted in a one-to-one dialogue with the respondents in the case of Datoga and Hadza; questions were read aloud and explained if necessary. Russian students completed demographic data and the aggression questionnaire individually. Institutional approvals, including Moscow State University Ethical Committee (MSUEC), and local governmental agencies (including Commission for Science and Technology, COSTECH and National Institute for Medical Research, NIMR of Tanzania), were obtained prior to conducting this study. All subjects gave their informed, verbal (in the case of Datoga and Hadza) and written (Russians), consent prior to participation. Verbal consent was deemed appropriate given the low literacy rates in traditional Hadza and Datoga, and was approved by the MSUEC and the COSTECH.

The Hadza people are one of the few groups still living the traditional way of life [45, 46]. This is an immediate return society. Their culture in many respects remains the same as that was of Bushmen of Namibia until the 1970s [47–49]. Hadza live in the Eyasi Basin, near Lake Eyasi in northern Tanzania, and speak the click language (Hadzane) [50, 51].

The Hadza have the lowest heterozygosity within Africa, possibly due to severe population bottleneck effects undergoing both in the past and recently [52]. At present, the Hadza population approximates 1000 people. About 350 Hadza (part of Eastern Hadza) still live as traditional hunter-gatherers and have been subjected to strong selective pressure very similar to that characteristic of ancestral human populations in the Neolithic [53–56].

Hadza are known for their egalitarianism and relative peacefulness, with leadership being merely nominal [46, 48, 49, 55, 56]. On the other hand, if Gardner’s [57] classification of cultures were to be applied, Hadza’s culture would be classified as individualistic. Individuals are little restricted in their behavior; they are able to express their dissatisfaction, verbal aggression, and anger quite openly and, if in conflict with other people in the camp, may easily leave. Hadza do not have strict cultural taboo against verbal aggression or hostility towards strangers (our personal observations). The Hadza do not estimate aggressiveness as positive individual feature, at least, both sexes in this culture rate calm character as an important factor for their marriage partner choice [58]. On the other hand, they can behave aggressively, loudly quarreling with each other, and even fight over resources; fight and verbal aggression may occur during disputes between spouses [35].

The Datoga are semi-nomadic polygynous pastoralists; the population size approximates 90,000. They are patrilocal and patriarchal. In contrast to the egalitarian and relatively peaceful Hadza foragers, the Datoga who live in the same region [44, 59, 60] have been selected for their aggression, particularly their total aggression, as have other African pastoralists [61, 62]. As in the case of Hadza, Datoga use various tapes of aggression, physical, as well as verbal. Thus is true for both sexes. While men and women may fight with same sex opponents, aggression between spouses is highly asymmetrical, being directed exclusively on women [44]. Men with fierce characters and warrior skills may be more successful in their mating and parental efforts because they can acquire cattle while raiding and in turn may better protect their own cows. Furthermore, our data suggest a possible link between total aggression and direct fitness caused by strong sexual selection in Datoga men, while such a relationship most likely became vague in Westernized societies [39].

Russians in this study was represented by university students from Moscow, and as it was earlier reported, the rate of physical aggression in this sample was low, but the usage of verbal aggression was deemed quite appropriate [63].

### Genotyping

Buccal epithelium samples were collected from all participants for DNA analysis. Genomic DNA was isolated using the Diatom DNA Prep 200 extraction kit (IsoGeneLab, Russia).

Polymorphic variants of the genes *HTR1A*, *HTR2A*, and *5HTTLPR* were analyzed after locus-specific polymerase chain reaction (PCR) with a kit of GenePak® PCR MacterMix Core (IsoGeneLab) according to the manufacturer’s protocol. For details of the sequences and annealing temperatures of the primers, as well as the restriction endonucleases, see Table 2 [64, 65]. The amplification conditions included initial denaturation at 94 °C for 4 min and 35 cycles consisting of three steps: denaturation for 1 min at 94 °C, primer annealing for 1 min at *х* °C, and elongation for 1 min at 72 °C. The last step included final elongation for 2 min at 72 °C. To identify the SNP, the amplification products were divided into equal aliquots of 10 μL, one of which was treated with the appropriate restriction endonuclease (5 units per sample) at *T*_opt_, °C overnight. The amplification and restriction products were fractionated in 2% agarose gel with ethidium bromide staining. The results were analyzed and photographed on a BioDocAnalyze device.

**Table 2:** Analyzed section, primer sequences, amplification conditions, and restriction endonucleases

Gene, locus	Primer sequences	Primer annealing temperature	Restrictase, *T*_opt_
*HTR1A*, rs6295	F: 5′-ggctggactgttagatgataacg-3′ R: 5′-ggaagaagaccgagtgtgtcat-3′	59 °С	*Bse*GI, 55 °C
*HTR2A*, rs6311	F: 5′-atattgaaggcatgagagtggttga-3′ R: 5′-tttttaggctgaagggtgaagaga-3′	56 °С	*Msp*I, 37 °C
*5-HTTLPR*, VNTR, rs25531	F: 5′-gcgctcctgcatcccccatta-3′ R: 5′ gggatgcgggggaatactggt-3′	59 °С	*Msp*I, 37 °C

### Statistical analysis

Prior to the search for associations, genetic structure of the samples studied for the three genes was revealed. GenAlEx software v 6.503 was used to calculate genotype and allele frequencies, heterozygocities, and the Hardy–Weinberg equilibrium (HWE). The differences were considered statistically significant at *p* < 0.05. Benjamini and Hochberg [66] correction was used for multiple comparisons.

All data were tested using one-way ANOVA, and multivariate ANOVA by means of SPSS-20 software. The level of significance was set at *p* < 0.05.

## Results

In the samples of Hadza, Datoga, and Russians, three 5-*HTTLPR* and two 5*HTR1A* and 5*HTR2A* alleles were identified. The data on the allele distributions at these loci are presented in Table 3.

**Table 3:** Allele frequencies at the three genes in the three samples studied

Locus	Allele/n	Hadza	Datoga	Russians
5-*HTTLPR*	N	327	319	205
	*Sa*	0.191	0.187	0.461
	*Lg*	0.209	0.127	0.046
	*La*	0.599	0.687	0.493
*HT1A* rs6295	N	327	319	205
	*C*	0.240	0.503	0.471
	*G*	0.760	0.497	0.529
*HT2A*, rs6311	N	327	319	205
	*G*	0.665	0.607	0.620
	*A*	0.335	0.393	0.380

The most frequent alleles were *La* (5-*HTTLPR*), *G* (5*HTR1A*), and *G* (5*HTR2A*). The distribution of 5-*HTTLPR* genotypes were in Hardy-Weinberg equilibrium in two populations (*χ*^2^ = 2.28, d.f. = 3, *p* = 0.517 (q* < 0.017), for Hadza, and *χ*^2^ = 0.90, d.f. = 3, *p* = 0.825 (q* < 0.017), for Russians), and HWE was significantly broken in Datoga (*χ*^2^ = 23.77, d.f. = 3, *p* = 0.00003 (q* < 0.0003). The distribution of 5*HTR1A* genotypes were in HWE in two populations (*χ*^2^ = 0.38, d.f. = 1, *p* = 0.537 (q* < 0.017), for Datoga, and *χ*^2^ = 0.16, d.f. = 1, *p* = 0.689 (q* < 0.017), for Russians) and significantly broken in Hadza (*χ*^2^ = 8.91, d.f. = 1, *p* = 0.0028 (q* < 0.0033)). The distribution of 5*HTR2A* genotypes were in HWE in the all three populations (*χ*^2^ = 3.62, d.f. = 1, *p* = 0.057 (q* < 0.017), for Hadza; *χ*^2^ = 1.05, d.f. = 1, *p* = 0.689 (q* < 0.017), for Datoga; and *χ*^2^ = 3.92, d.f. = 1, *p* = 0.048 (q* < 0.017), for Russians).

The test for heterogeneity in the allele distributions between three samples showed highly significant pairwise differences for the 5-*HTTLPR* gene (*G* = 17.0 ÷ 119.2, d.f. = 2, *p* = 1.99E−04 ÷ 1.30E−26, Benjamini and Hochberg correction for the three pairwise comparison: q* < 0.05); highly significant differences for the 5*HTR1A* gene for the following pairs Hadza-Datoga, *G* = 97.4, d.f. = 1, *p* = 5.77E−23, q* < 0.033, and Hadza-Russians, *G* = 60.1, d.f. = 1, *p* = 9.20E−15, q* < 0.033, while Datoga-Russians had almost the same allele distribution, *G* = 1.1, d.f. = 1, *p* = 0.306, q* < 0.033; and nonsignificant differences for the 5*HTR2A* gene for all three pairs, G = 0 ÷ 4.8, d.f. = 1, *p* = 0.03 ÷ 1.0.

The data were obtained for all four scales of BPAQ (29 questions). Because verbal aggression was considered acceptable in all the three study groups, we considered important to include the ratings on this subscale as well. For the purposes of this paper, we targeted the total aggression (represented as sum these scales), because we were intended to target aggression per se. In doing this, we account for possible cultural differences (particularly, cultural restrictions in applications of physical aggression, anger, and hostility). The association analysis for total aggression was conducted separately in each of the studied population. The following Cronbach’s alphas were obtained: Hadza, 0.79; Datoga, 0.79; and Russians, 0.88. Thus, the reliability may be interpreted as sufficient (Hadza and Datoga) and good for Russian sample.

The data on self-ratings on total aggression based on BPAQ questionnaire for men and women in three studied samples are presented in Table 4. We did not find significant differences on total aggression scores between sexes in all three studied samples. The total aggression scores for the three samples studied are as follows: Hadza—83.23 ± 0.91, Datoga—97.77 ± 0.92, and Russians—79.60 ± 1.19. We conducted one-way ANOVA to compare the ratings on total aggression between the three studied samples, and the differences were significant (*F* = 95.33, d.f.1 = 2, d.f.2 = 850, *p* < < 0.001). Post hoc tests revealed significant differences for all three pairs compared (Hadza-Datoga—*p* = 0.0001; Hadza-Russians—*p* = 0.04; Datoga-Russians—*p* = 0.0001).

**Table 4:** Mean scores on total aggression in three populations and sex differences in each population

Population	BPAQ	Men (M ± Std.Err.)	Women (M ± Std. Err.)	One-way ANOVA
Hadza	Total Aggression	84.52 ± 1.15	81.57 ± 1.45	*F*_(1,327)_ = 2.62, *p* = 0.107
Datoga	Total Aggression	99.23 ± 1.16	96.25 ± 1.42	*F*_(1,317)_ = 2.65, *p* = 0.105
Russians(students)	Total Aggression	81.70 ± 1.82	78.04 ± 1.56	*F*_(1,203)_ = 2.27, *p* = 0.134

*M* mean value, *Std. Err* mean standard error

**Fig. 1:**
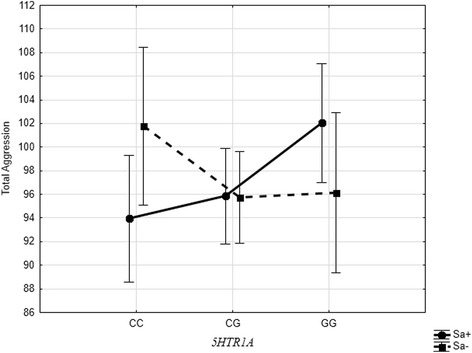
The interaction effect of the *5-HTTLPR* and *5HTR1A* polymorphisms on self-rating scores of total aggression in Datoga

Because we found the distributions of allelic frequencies for 5-*HTTLPR* and 5*HTR1A* genes were significantly different between the three samples, it was impossible to conduct the general analysis for the pooled data. Besides, due to the fact that Russian sample contained the data on one age group only, we decided not to include age variable in the equation to make data processing identical. Consequently, we developed the following model for each sample separately: a three-way ANOVA (μ + *5-HTTLPR* + 5*HTR1A* + 5*HTR2A* +ε) with measures of self-reported total aggression as dependent variable (Tables 5, 6 and 7). We did not include sex variable in the model, as there were no sex differences for the total aggression scores in all three samples. Both main and two-way interaction effects of the three polymorphisms were included in the ANOVA model. The only significant results were obtained for the Hadza sample; the main effect of the second serotonin receptor polymorphism appeared to be significant (post hoc: *AA*-*AG*, *p* = 0.001; *AA*-*GG*, *p* = 0.002; *AG*-*GG*, *p* = 0.660). For Datoga, the interaction effect between *5-HTTLPR* and 5*HTR1A* was significant (Fig. 1). As follows from Fig. 1, there is significant difference between *Sa+* genotypes and *LaLa* genotype in association with the *CC* genotype of the 5*HTR1A*, but not in association with the *CG* and *GG* genotypes. No significant effects of the studied polymorphisms were obtained for Russians.

**Table 5:** The results of three way ANOVA with interactions between three genes

Hadza					
Source of variation	D.F.	SS	MS	*F*	*p*
5-*HTTLPR*	1	201.62	201.62	0.78	0.377
*5HTR1A*	2	334.02	167.01	0.65	0.524
*5HTR2A*	2	1718.86	859.42	3.33	0.037
5-*HTTLPR × 5HTR1A*	2	386.25	193.12	0.75	0.474
5-*HTTLPR × 5HTR2A*	2	554.05	277.03	1.07	0.343
*5HTR1A × 5HTR2A*	4	391.64	97.91	0.38	0.823
Error	315	81,223.24	257.85		

*R*^2^ = 0.084

*D.F* degrees of freedom, *SS* sum of squares, *MS* mean square, *F* Fisher criterion, *p* probability value

**Table 6:** The results of three way ANOVA with interactions between three genes

Datoga					
Source of variation	D.F.	SS	MS	*F*	*p*
5-*HTTLPR*	1	7.88	7.88	0.29	0.864
*5HTR1A*	2	356.84	178.42	0.66	0.516
*5HTR2A*	2	482.81	241.41	0.90	0.409
5-*HTTLPR × 5HTR1A*	2	1697.40	848.70	3.15	0.044
5-*HTTLPR × 5HTR2A*	2	243.02	121.51	0.45	0.637
*5HTR1A × 5HTR2A*	4	152.91	38.23	0.14	0.966
Error	305	82,155.76	269.36		

*R*^2^ = 0.039

*D.F* degrees of freedom, *SS* sum of squares, *MS* mean square, *F* Fisher criterion, *p* probability value

**Table 7:** The results of three way ANOVA with interactions between three genes

Russians					
Source of variation	D.F.	SS	MS	*F*	*p*
5-*HTTLPR*	1	698.67	698.67	0.69	0.406
*5HTR1A*	2	672.11	336.06	1.17	0.312
*5HTR2A*	2	1250.42	625.21	2.18	0.116
5-*HTTLPR × 5HTR1A*	2	312.96	156.48	0.55	0.580
5-*HTTLPR × 5HTR2A*	2	840.27	420.13	1.7	0.234
*5HTR1A × 5HTR2A*	4	332.82	83.21	0.29	0.884
Error	191	54,787.11	286.84		

*R*^2^ = 0.072

**Fig. 2:**
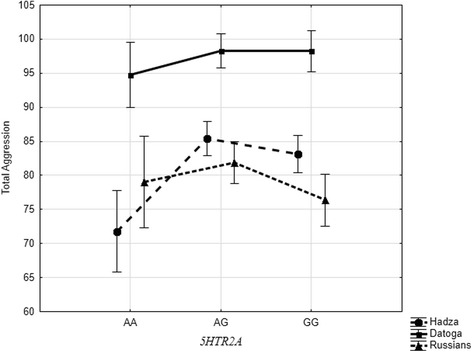
Main effects of ethnic, *5HTR2A* polymorphism, and their interaction on self-rating scores of total aggression

The results of two-way ANOVA with ethnicities and the 5*HTR2A* polymorphism as main effects and their interactions revealed the highly significant effect of ethnicity, 5*HTR2A* polymorphism, and their interaction on total aggression (Table 8, Fig. 2). As follows from Fig. 2, there is highly significant difference between Datoga and the other two ethnics in scores on total aggression in association with the all three 5*HTR2A* genotypes. Russians and Hadza differ significantly in total aggression only in association with the *GG* genotype.

**Table 8:** The results of two-way ANOVA with ethnicities and the *5HTR2A* polymorphism as main effects and their interactions

Source of variation	D.F	SS	MS	*F*	*p*
Ethnic	2	42,032.45	21,016.42	78.22	0.0001
*5HTR2A*	2	3627.26	1828.63	6.81	0.001
Ethnic *× 5HTR2A*	4	2795.36	698.84	2.60	0.035
Error	844	226,762.69	268.68		

*R*^2^ = 0.206

*D.F* degrees of freedom,k *SS* sum of squares, *MS* mean square, *F* Fisher criterion, *p* probability value

## Discussion

The first prediction was supported by our data, the differences in total aggression scores between samples were significant, and the total aggression being the highest for Datoga and the lowest for Russian sample, and Hadza rated in between. Thus, we conclude that the cultural factor does matter and should be considered obligatory in all gene-environment studies. On the contrary, the second prediction was not supported and sex differences in total aggression were not found in any of our study samples. In line with our third predictions, differences allele frequency distributions between populations were found for two polymorphisms, 5-*HTTLPR* and *5HTR1A*, but absent for the third one, *5HTR2A*.

The main effect of 5-*HTTLPR* polymorphism on the total aggression was not observed in any of our study groups; thus, the fourth prediction was not supported. We did not find any main effect of *5HTR1A* polymorphism either; thus, our fifth prediction failed. The important finding of this study is the association of *G* allele of *5HTR2A* polymorphism with higher total aggression within each sample. Thus, the sixth prediction was supported. We did not find any interaction effects for the combination 5-*HTTLPR* and *5HTR2A*, as well as for *5HTR1A* and *5HTR2A*. The interaction effect appeared significant for the combination 5-*HTTLPR* and *5HTR1A*, but only in one study sample, particularly, in Datoga. Hence, our last prediction was partly supported.

Concerning the polymorphisms targeted in this study, the allele distribution of the *5-HTT* gene for the Russian sample was in good correspondence with the Caucasian sample, previously studied by other authors (North Americans of the European origin) Wendland et al. [67]. Both African samples differed from the Caucasian sample in the allele frequency distributions: they had lesser amount of *Sa* allele and higher amount of *Lg* allele.

In studies of Canadian samples from Quebec and Ontario and Japanese sample [68, 69] of the rs6295 (5*HTR1A* gene), polymorphism prevailing allele was *С*, which frequency varied from 0.63 to 0.84, while in our populations the maximum of 0.50 for the *С* allele was observed in Datoga.

Our study demonstrated also the prevalence of the *G* allele of the 5*HTR2A* gene, 0.61 in Datoga and 0.66 in Hadza with the Russians in between, which is in a good agreement with results on Caucasians from the USA and Australia, and Japanese people [70, 71].

The data on BPAQ self-ratings demonstrated that the Datoga rated significantly higher on total aggression than other two groups. These data are interesting from the point of cultural anthropology to the extent that Datoga are seminomadic pastoralists [72], polygynous, and horizontally divided into generation sets with clear wealth stratification [36, 73]. Traditionally, Datoga have to be well trained to use aggression to protect their herds from raids of other ethnics, as well as wells and pastures from other Datoga [38]. Well possible that such differences are due to differences in socialization for aggression in three populations, particularly, the Datoga parents being more aggressive towards their children (B.M., personal observations). The egalitarian Hadza, who are still traditional [37], rated much less on total aggression compared with Datoga, which was in line with our expectations, but still, they rated higher compared to Russians (represented in our study by students). Obviously, in Hadza, as well as in modern Russian culture, total aggression is under social control and children are socialized against total aggression since early childhood. Thus, the differences in aggression scores between the Datoga and other two groups were expectable.

The results of ANOVA demonstrated significant effects of population origin and *5HTR2A* polymorphism on self-ratings on total aggression. Carriers of *AG* and *GG* genotypes of *5HTR2A* rated significantly higher than *AA*. Previously, it had been suggested that the 5*HTR2A* gene polymorphisms associate with aggression, criminality, hostility and anger [3, 74–77], and antisocial behavior [78, 79], or maladaptive impulsivity [80]. Our data extends these findings, demonstrating the role of rs6311 *5HTR2A* in total aggression in adult healthy men and women from traditional African societies, as well as for Russian student sample.

Our data demonstrate the importance of cross-population comparisons in gene-environmental studies. While the significant interaction effect of *5-HTTLPR* × *5HTR1A* was obtained for Datoga, we did not get any significant associations in Hadza and Russians. Possible explanation is that socialization for aggression was different in these cultures: relatively tolerant (or even simulative) in Datoga and suppressive in two other cases. Thus, our findings provided further confirmation about important role of cultural factors mediating gene-gene-environment interactions, thus adding to the results of other studies [19].

Our study has certain limitations, first of all, because we presented the data on candidate gene association study (CGAS). It is currently obvious that genetic factors involved in aggressive behavior are likely to have a small effect size, and environmental factors may influence substantially to their expression [9]. The results obtained by different authors using CGAS approach are often contradictory, or not replicable, since in several cases associations were identified with different alleles of the same genetic variant. Thus, our results on association of rs6311 *HTR2A* polymorphism with total aggression in adults should be taken with caution. Still, it is important to mention that our data were obtained for healthy adults from three populations, are quite representative (*n* = 853), and were collected using the identical protocol. These data demonstrated the need of careful investigation of investment of social environmental data, as well as sex differences in total aggression along with association between gene polymorphisms and particular behavioral trait. We suggest that *5HTR2A* gene should be further investigated in the context of gene-environmental studies on aggression along with other genes involved in neurotransmitters and hormonal functions. Given the current data on genome-wide studies in aggression, it is also reasonable to consider potential risk genes and pathways involved in neurodevelopmental processes, including neuron projection and synaptic plasticity [9]. Other limitations, which need to be mentioned, concern our Russian study sample, as contrary to other two groups, it was represented only by students. Thus, not all age groups were presented, besides, not all social strata included. Still, we suggest, that this sample was more or less representative for the whole population, given the fact that we were not working with elite Universities, but collected data in different universities, with students, coming from families, belonging to different social classes. Future studies are needed to shed the light on this point.

## Conclusions

Our data provided obvious confirmation for the necessity to consider the population origin, as well as cultural background of tested individuals, while searching for associations between genes and behavior, and demonstrated the role of cultural attitudes towards the use of in-group aggression. Our data partly explained the reasons for disagreement in results of different teams, searching for candidate-gene associations with behavior without considerations of culturally desirable norms. Previous studies suggested that the 5*HTR2A* gene polymorphism associates with aggression and criminality. Our data extended these findings, demonstrating the role of rs6311 (5*HTR2A* gene) in aggression in adult healthy men and women from our samples. We found that *G*-allele carriers were rated higher on total aggression.

## Abbreviations

5-HT: 5-Hydroxytryptamine or serotonin
5HTR1B: 5-Hydroxytryptamine Receptor 1B gene
*5*HTR2A: 5-Hydroxytryptamine Receptor 2A gene
*5HTTLPR*: *Serotonin-transporter-linked polymorphic region*
ANOVA: Analysis of variance
*AR*: *Androgen receptor gene*
*AVP*: *Arginine Vasopressin gene*
BPAQ: Buss and Perry’s Aggression Questionnaire
CGAS: Candidate gene association study
*DAT*: *Dopamine transporter gene*
*DRD2*: *Dopamine receptor D2 gene*
*DRD4*: *Dopamine receptor D4 gene*
*ESR1*: *Estrogen Receptor 1 gene*
GLM: Generalized linear model
HWE: Hardy–Weinberg equilibrium
*MAOA*: *Monoamine oxidase A gene*
*OXTR*: *Oxytocin receptor gene*
SNP: Single nucleotide polymorphism
